# Navigating and making sense of urgent and emergency care processes and provision

**DOI:** 10.1111/hex.12866

**Published:** 2019-01-10

**Authors:** Catherine Pope, Gemma McKenna, Joanne Turnbull, Jane Prichard, Anne Rogers

**Affiliations:** ^1^ NIHR CLAHRC Wessex University of Southampton Southampton UK; ^2^ Health Sciences University of Southampton Southampton UK

**Keywords:** citizen panel, emergency care, interview, sense‐making, urgent care

## Abstract

**Background:**

Whilst many health systems offer a range of urgent and emergency care services to deal with the need for unscheduled care, these can be problematic to navigate.

**Objective:**

To explore how lay people make sense of urgent care provision and processes.

**Design:**

Qualitative study, incorporating citizen panels and longitudinal semi‐structured qualitative interviews.

**Setting and Participants:**

Two citizens’ panels, comprising purposively selected public populations—a group of regular users and a group of potentially marginalized users of urgent and emergency care. Semi‐structured interviews were conducted with 100 people, purposively sampled to include those over 75, aged 18‐26 years, and from East/Central Europe. A sub‐sample of 41 people received a second interview at +6‐12 months. Framework analysis was thematic and comparative, moving through coding to narrative and interpretive summaries.

**Findings and Discussion:**

Participants narratives illuminated considerable uncertainty and confusion regarding urgent and emergency care provision which in part could be traced to the contingent nature of urgent and emergency care need. Accounts of emergency care provision were underpinned by strong moral positioning of appropriate help‐seeking, demarcating legitimate service use that echoed policy rhetoric, but did not necessarily translate into individual behaviour. People struggled to make sense of urgent care provision making navigating “appropriate” use problematic.

**Conclusions:**

The focus on help‐seeking behaviour, rather than sense‐making, makes it difficult to move beyond the polarization of “appropriate” and “inappropriate” service use. A deeper analysis of sense‐making might shift the focus of attention and allow us to intervene to reshape understandings before this point.

## 1 INTRODUCTION

In developed countries, including the UK, USA, Canada and Australia, urgent health‐care services are often positioned in an ill‐defined space somewhere between family or general practice (GP) and emergency hospital and ambulance care.^1^, ^2^, ^3^ Urgent health‐care services are primarily designed to assess and manage unscheduled or unforeseen conditions that arise in the out‐of‐hours period, providing care for people with pressing health‐care needs which cannot wait until primary care services are available.^4^ The nomenclature and branding varies across health systems, as does the scope and content of these services. English NHS urgent health care has expanded to include a range of services (GP out‐of‐hours, walk‐in centres, urgent care centres, minor injuries units and a national telephone helpline for out of hours urgent care NHS 111).^1^, ^5^, ^6^, ^7^ Services often overlap, and are sometimes co‐located with, emergency care departments (EDs), or with primary care provision including pharmacies, or social care. Urgent care services reflect a succession of shifts in approaches to health‐care provision and so are increasingly fragmented and differentiated, reliant on new technologies such as computerized triage systems and digital record keeping.^5^, ^8^ Descriptions of the services often reflect the consumer‐focus and patient choice mantras of recent health policy.^9^

The boundary between urgent and emergency health care and other health‐care provision is a site of tension and attention. Urgent care has become a strategic focus for managing demand, with the aim of diverting people away from overburdened emergency health‐care services.^1^, ^10^ The Keogh review^1^ outlined a vision for future urgent and emergency services in the UK in which people would be better supported to self‐care, but could access urgent care via NHS111 as the main point of entry into the urgent care system. This vision was represented as an inverted pyramid in which most people self‐care or access a range of urgent care services such as GPs, urgent care centres, community nurses or pharmacists, with only the more serious or life‐threatening conditions requiring access to the specialist services of hospitals and emergency departments. Emergency care is expensive relative to primary health services^11^, ^12^ strengthening the case for more use of, apparently cheaper, urgent health‐care services and more self‐care advice. This is supported by evidence that overcrowded EDs may increase delays to receiving treatment, and add to discomfort, anxiety and burdens on patients seeking help.^13^, ^14^ The discourse surrounding urgent and emergency health‐care echoes these concerns, focusing on claims that 12% to 40% of attendances are “inappropriate,”^15^ and figures that suggest that some 40% of patients are discharged from the ED without treatment.^1^

Navigating (identifying and connecting with the relevant options) between urgent and emergency health care and other services may be confusing and complex for individuals seeking, or considering, help‐seeking. People are required, often at the point when sick or injured, to distinguish between health‐care needs that are categorized as “routine,” “urgent,” “emergency,” “primary” or “acute” and are confronted by an array of possible services, to which access may vary according to time of day, and day of week. There is some suggestion that a key driver of ED attendance is lack of access to primary care services^16^, ^17^ which may be a factor driving urgent care demand to EDs. However, a recent qualitative study exploring why patients choose to attend the ED suggested experiential knowledge of previous service use might be more relevant in decision making^18^ suggesting that people are not merely applying categories when making decisions to seek help.

The concept of sense‐making can be enrolled to inform thinking around health‐related help seeking. Prior to making decisions people draw on existing representations of their knowledge and beliefs around illness and about the health‐care provision available to them and integrate these with their current circumstances to make sense of the situation. This might be done alone or through contact with their wider social network. Weick^19^ suggests that sense‐making can be understood as the manner by which people enact their environment. It is a process requiring interaction with people and objects as a means of articulating the unknown in an attempt to make sense of a complex set of circumstances by turning these, “into a situation that is comprehended explicitly in words and serves as a springboard to action.”^19^ Sense‐making thus can be seen variably as a cognitive information processing activity^20^ and as a social process.^21^

This paper presents a detailed exploration of the lay experiences, perceptions and sense‐making surrounding the boundaries and utilization of urgent and emergency care. It begins with a brief overview of UK policy and relevant research to illuminate some of the core definitions surrounding urgent and emergency health‐care services and it is presented as a context for data considered in two citizens’ panels exploring lay members’ conceptualizations of urgent and emergency care services. These data are augmented by analysis of 141 interviews with lay people exploring in detail their sense‐making with regard to urgent care. Together these data help to demarcate a distributive struggle^22^ that characterizes the tensions and challenges of help‐seeking, “over use,” and “inappropriate attendance” that occur when users encounter and think about the use of urgent and emergency health care at the interface with other service provision.

### 1.1 Defining urgent and emergency NHS care in policy and research literatures

**Figure 1:**
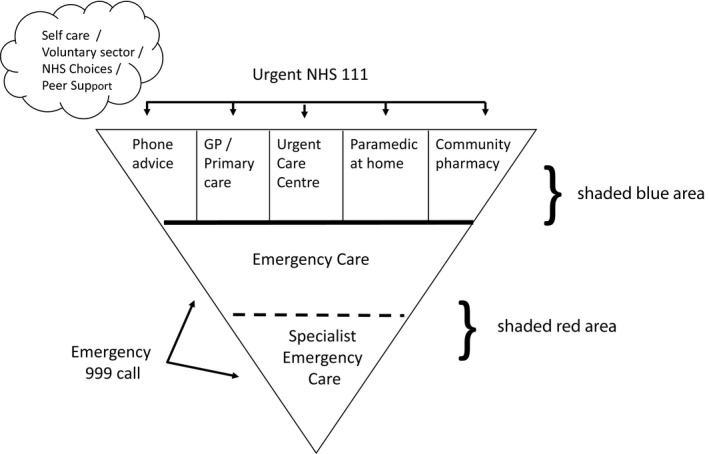
Keogh model of urgent and emergency care, adapted from NHS England^7^ (p8)

The Urgent and Emergency Care Review^1^, ^4^, ^7^ presents a pyramid model of services (Keogh model) which are distinct from one another and provide for varying levels of need (see Figure 1).

In these policy documents, emergency and urgent care needs are defined by reference to their own labels and to each other; urgent is compared to emergency as “not life‐threatening,” and designated as “serious” versus “more serious” emergency presentations.^1^, ^4^, ^7^ Thus, “urgent” conditions may be described as “serious but not life‐threatening”^4^, ^23^ and urgent care services “for people who feel urgently ill” (p. 37).^24^ There are hints of the model of services being based on a hierarchy of need, but no real explanation of how the boundaries between services are operationalized. Few policy documents provide a working definition of urgent or emergency health‐care needs. There is a vaguely specified suggestion that the designation of urgent or emergency hinges on the speed with which a person needs to be seen.^24^, ^25^ Some policy documents conflate urgent and emergency care services into a single category, labelled as unplanned or unscheduled care^4^ thus avoiding the idea of a boundary altogether.^26^, ^27^ Often policy makes no reference to a definition at all.^7^, ^25^, ^28^, ^29^, ^30^, ^31^

Implicit in these characterizations is the idea of borders between services, determined by acuity, but it is unclear how these are established. The notion of urgency is contested and it is unclear who has the right to categorize it: it can be determined by service providers, users, or both.^6^ However, there is growing recognition that patients are less able to distinguish between services, precisely because of the confusion about terminology and definitions^26^ and the policy literature sets up a hierarchical model of urgent and emergency care which lacks specificity and offers little traction for those navigating these services.

The academic literature is similarly unhelpful. Conceptualizations of urgent and emergency care are discussed in terms of appropriateness of service use, particularly in relation to ED and ambulance services. Inappropriate attendance includes cases deemed “low urgency” or “unnecessary,” with the suggestion that the patient could have been responded to elsewhere.^32^ Differences in professional perceptions of urgency and wide variance in what is considered as appropriate service use is also evident.^33^, ^34^ Quan et al^35^ found that professional assessment of urgency was based around timeframe and contextual subjectivity, such as whether the patients or their family was upset, rather than clinical features alone. Furthermore, definitions of urgency varied between physicians and nurses, with nurses more likely to take in the wider context of the patient experience. Koziol‐McLain et al^36^ suggest that the term “severity” is embedded in the “medical framework of physiologic dysfunction or disease” and they define emergency care as “those health services provided to evaluate and treat medical conditions of recent onset and severity” (p. 561). From this perspective, patients are seen to access care in response to bio‐medical crisis exclusively, with psychological and social factors not requiring consideration. In the context of a literature depicting a lack of clarity about borders and positionality of services, it seems sensible to explore how people make sense of urgent care provision and processes, and how this impacts on their navigation of services.

## 2 METHODS

To explore service users sense‐making about urgent care help seeking, we conducted citizens’ panels and interviews. Citizens’ panels are a method used to assess public preferences and opinions. They permit participants to “engage with evidence, deliberate and deliver recommendations on a range of complex topics.”^37^ We used a modified citizen panel approach, allowing participants to facilitate their own discussion, enabling a collaborative bottom‐up approach to consensus development.

Two general public panels were conducted which included people known to be more regular users of urgent and emergency care (such as parents of young children, older patients), and sought representation from different ethnic groups and geographical areas. We included adults aged 18 and over, and the oldest participant was 78 years of age. One panel consisted of people drawn from the East European community, chosen because this population is known to be growing in size in the chosen setting, and because more recent migrants may lack familiarity and experience with local services as a basis for sense‐making and may be a marginalized group.^38^ Participants were recruited via local community groups and networks (eg, via community centres), public advertising (posters, press and local radio) and local service providers. In total 24 participants took part in the citizens’ panels with 12 in each panel. Written consent was obtained from each panel participant. Panels were facilitated by two members of the research team, one acted as facilitator (GM), one in a supporting and observational role taking notes (JT). The panels included structured activities to stimulate and guide discussion aimed at reaching a consensus using examples of urgent care definitions. We drew on web links, video and visual resources as prompts.

Semi‐structured interviews were conducted with a separate sample of people. Initial interviews explored how people distinguished between routine, urgent and emergency care needs, and understood available services. A second interview was conducted between 6 and 12 months later with a sub‐set of participants exploring items raised in the first interview in more detail and information about recent urgent or emergency care help‐seeking. We sampled from a geographical area covering four English counties and purposively sampled from three populations representing facets of urgent care need and socioeconomic and demographic characteristics. Two groups were chosen to reflect populations with known high use of emergency care (people aged 75+ years and those aged 18‐26 years) and a third group, people from East and Central European communities following up on themes generated in the citizens’ panels about the experiences of these migrant populations.

Recruitment to interviews took place between September 2016 and July 2017. We had anticipated that participants would be recruited via NHS urgent and emergency care services however this proved very difficult (only 13 participants were recruited in this way). We therefore widened our strategy and recruited a further 87 participants from the general population using community‐based advertising and local media advertising to meet sample targets. Interested participants were either sent an information pack by e‐mail or a research nurse handed them (at the ED or urgent care centre) or posted an information pack (NHS 111 and community sample). To encourage greater uptake of interviews, we offered a £15 gift voucher (per interview) as an incentive to take part. We conducted 93 first interviews with 100 people (some in pairs, usually older couples where a spouse or partner was present in the home when the interview took place). All participants were invited to take part in second interviews. In total, 41 participants were available and agreed in take part in a second interview. Interviews were conducted by two female members of the research team (GM and JT) and lasted between 35‐90 min. The interviews were digitally recorded and transcribed as anonymized documents for analysis by the wider team.

### 2.1 Analysis

Data generated included written notes, audio recordings of group discussions which were later transcribed, and written material produced by panel members including post‐it notes, flipchart lists and diagrams. Data analysis initially focussed on the text and visual data generated during the citizens’ panels and took place throughout the collection of both panel and interview data. We undertook a thematic analysis of these data following the stages described by Braun and Clarke (2006),^39^ familiarising ourselves with the data, generating initial codes and categories and then identifying themes. Data from the citizens’ panels were compared to examine the similarities and differences between panels. Visual data, notably, the panels’ re‐drawings of the Keogh model, but also other material on flip charts and post‐it notes, were included in the analysis, as were notes audio recordings.

Qualitative interview data were analysed using a team approach to share and interpret data collectively, building emergent themes and developing narrative and interpretive summaries. The research team read and open coded a sample of transcripts and panel reports independently, discussed emerging codes to form the basis for a coding scheme which was refined and applied to all transcripts. We drew on the Framework Analysis approach^40^ to aid comparative analysis, in particular to identify factors that appeared common or different across different data sources and different population groups (younger/older/East and Central European groups). Analyses were informed by conceptual ideas on sense‐making and to facilitate analysis and discussion amongst the team, grids and matrices were used to chart and compare the data. Emerging themes were shared with the wider research team comprising the fieldworkers, researchers and clinicians and discussed with advisors including patient representatives to check credibility and refine thinking.

## 3 FINDINGS AND DISCUSSION

Sense‐making can be understood as activity by actors in an organization or system, including an open system such as patients systems of personal communities or networks, in which attempts to structure the unknown are made by cognitive activity^41^ such as placing information into “frameworks, comprehending, redressing surprise, constructing meaning, interacting in pursuit of mutual understanding, and patterning” (p6).^20^ Our analysis explored understandings of the health‐care system and meanings to users of services. We present the findings under four thematic headings (a) confusing boundaries of urgent care service provision, (b) contingent nature of need, (c) moral positioning in making sense of when and how to use urgent care and (d) re‐imagined borders of urgent and emergency provision.

### 3.1 The confusing boundaries of urgent care service provision

Services identified as potentially available for urgent and emergency care included expected answers such as general practice, ambulance services (contacted via the 999 telephone number) and emergency departments, NHS walk‐in centres, pharmacies and the NHS 111 helpline. However, reference was also made to an extended network of specialist services ‐mental health, end of life, hospice care, geriatric medicine, physiotherapy and dental services, information and advice services and non‐health‐care services including social services, police and patient transport. Previous research^42^ prior to the setup of the system of NHS walk‐in centres suggested that potential users made sense of them by framing expectations with reference to the configuration of General Practice provision. Exploring a different set of users’ perspectives 17 years later, there was less clarity and more confusion among our participants when it came to specifying in more detail what constituted urgent care centres and minor injuries units. Some people regarded the latter as “another name for A&E” (Accident and Emergency, a synonym for ED), others simply struggled to understand them:

P30: Walk‐in seems so casual. Pop in and out if you want. But urgent, urgent care centre makes it seem … If I were to see those two things and you would say to me do you think these two are, you know synonymous or do you think they are, you know two totally opposite things I would probably say a walk‐in centre and an urgent care centre seems two different … just by the words that are in the names … And then what was the third one?

Interview: A minor injuries unit?

P30: No clue. I have no clue. Can you tell me? (Younger interviewee)

Data analysed from East European panel respondents offered a restricted list of services under the umbrella of urgent and emergency care but noted that access to an on‐call doctor was important (this was something that recent migrants expected from prior experience of direct access to doctors in their previous homeland). These panel members were surprised to learn that community pharmacies were considered by policy makers to be part of the urgent care network of services, while the general public panel thought that pharmacies offered advice for “little ailments” and were a place to “seek a second opinion” rather than occupying a clear position within urgent care services. Thus for all participants, pharmacies played little part in their conceptualizations of urgent care services despite current evolving UK policy (exemplified by the Keogh first stage review^1^) that the skills, experience and accessibility of community pharmacists should be harnessed as part of future urgent and emergency care provision.

In contrast to intentions of current policy many services were perceived as equivalent rather than hierarchical or distinct, and, as others researchers have suggested, the distinctions between them were flexible, ambiguous and confusing^26^, ^30^:

We had a conversation here, didn't we, about the confusion, and how do you know what to do. And actually, you know, if you've used services a lot you know what to do. But if you've had an urgent care incident, and you've only had one in the last 20 years, how do you know what to do? (Public panel)

P14: Urgent care, I would think of, probably, well, an ambulance, A&E, you know, if it was urgent, yes. Otherwise it would be just a trip up the doctors to see what the problem is, you know.

Interviewer: … emergency care, what do you think of?

P14: Emergency care is, well, the same thing, really. Yes. I mean, if I could see there was a major problem with anything … if it really looked bad, you'd have to ring 999, I think. (Younger interviewee)

People understood “emergency” services as those designed for more serious, or life‐threatening conditions, but at times struggled to identify what might fall into these categories and frequently used the word “urgent” to describe such health‐care needs. Occasionally interviewees revealed a lag in understandings of the changing terminology for Emergency Departments (EDs). An older interviewee did not consider emergency care to include “Accident and Emergency” (a commonly used term for the ED).

P28: Emergency care, probably [for] something a bit less severe than A&E, but… Again, there's no real fine line, is there?  (Older interviewee)

Another interviewee, whose sense‐making of the UK system was shaped by understanding of a different system, reported that both “urgent” and “emergency” could apply to emergency services.

P3: Yes, [urgent might be] something that can't wait for very long or maybe can wait longer than emergency [ ] in … Polish I think we don't have separate words for these two. Maybe that's why… It's language problem as well, but in Polish, emergency and urgent … urgent sounds pretty serious. So maybe that's why we struggle to distinguish. I don't know. (East European interviewee)

### 3.2 Contingent nature of need

Making sense of the proximate types of care provision for urgent and emergency care was linked to a focus on time—an aspect mentioned in some UK policy.^24^, ^25^ “Urgent” need required “being seen there and then,” “immediately,” “instantly” or “quickly.” However, when asked to suggest alternative definitions of the terms urgent and emergency, the panel struggled to articulate how time could be factored into thinking about need for health care:

P1: If there's a certain target once you put a time limit on it, that's then a target.

[overtalking] P2: Yes. Then … P1: I mean that would have to make that a time period. Time in there, and we've got 24‐hour care. So, you're all saying to me is around timing. That's what we want to, to imply isn't it? You know, it's something that's as soon as possible. Requiring urgent care. (Public panel)

There was agreement that need alters over time as symptoms worsened, and that other factors influence need such as the vulnerability of the person needing help. Whilst a health‐care professional perspective might view need as contingent on risk, predisposing factors, age, and medical history, our lay participants struggled to place hypothetical cases in terms of this view of health‐care need, arguably lacking the expertise to do so. Contingencies in sense‐making were apparent, with some illustrative cases deemed to lie outside rules or norms further confusing boundaries between services. In common with previous sociological work^43^, ^44^ children and babies were considered special cases:

… a condition like chest infection wouldn't be urgent care matter for a very healthy 30 year old, but it would be very important for baby who is teething or sort of there's something… It needs to be tailored to the person and what their needs are and their mental health state and there's loads of things that need to be understood before you rate something as urgent or emergency or regular care. (Public panel)

We're a bit more worried about the toddler than anyone else. (Public panel)

In the absence of clearly defined boundaries of urgency, meaning was informed by factors such as existing beliefs, past experiences and understanding of the system in which possible actions are situated. Sense‐making of the urgent and emergency care system and its usage was maintained through the inclusion of “acceptable justifications” that allow for adaptations of the system.^34^ This struggle to make sense of the services on offer whilst also acknowledging contingencies and uncertainty surrounding health‐care needs underpins the third theme in our analysis—moral positioning.

### 3.3 Moral positioning in making sense of when and how to use urgent care

Our data illuminated how people judge and position other people and help‐seeking behaviours against moral principles entailed in making sense of what urgent and emergency care is for. Whilst service users described their own health service use and those close to them as legitimate, “others” were often characterized as “time wasters” and inappropriate service users. The quote below references a discourse rehearsing a moral position, and hints at contingencies that underpin perceived “illegitimate” help seeking:

P75: In hearsay, in stories, you sometimes hear about people who have got something absolutely piffling and yet they have gone to A&E or even called an ambulance.

Interviewer: And what would piffling be for you?

P75: You're coughing a lot or you have cut your finger on the tin opener or you have burnt your wrist on the oven shelf, you know really minor things, or you have got a temperature … a lot of people now apparently go to A&E because their child has croup and that I assume is because they have no idea what it is, and it is terrifying when you see it. (Younger interviewee)

Here an older interviewee brings in the confusion about services to make a similar claim:

P36: I'd like to know what priorities each service treats. I mean, some people must ring up 111 for a headache or something stupid like that. Well that should be made quite clear, that you go to your doctor if you've got a headache or any minor cut or anything like that. You don't ring them and waste their time because you get people who have had too much to drink and they've fallen over and they think, ‘oh well I'll ring the hospital or ring the walk in centre or whatever is available’. Whereas they could just as well wait until the next day. I feel very sorry for these people [health services] because they're overstretched all the time by a lot of idiots. (Older interviewee)

When asked to look at hypothetical cases panels discussed how contingencies might play into moral positioning. Social contingencies such as employment or access to transport are mobilized to legitimate attendance of others at the ED:

If she calls her employer and says, well I had to take my child to A&E, we were in hospital all night, I can't come to work, whereas if she goes like, yes, he was a bit poorly, he still has a temperature, I need to stay at home, she won't get the same, just, reaction from her employer…. There's a status thing about going to the A&E and… And needing that care. Sort of having all that, sort of forces people to go to… To sort of get validation or…? She can call her mum or her boyfriend and say, ‘oh, you know what happened? I had to go to A&E, and look at me’.(Public panel)

Other accounts rehearsed the language of policy and research literature concerning appropriate/inappropriate service use but were also informed by news reports of services under pressure, and reality television programmes about emergency care. The construction of moral categories echoes that performed by health‐care staff evident described previously by Davis and Strong^44^ and Roth.^45^ Sometimes derogatory terms were used —”frequent flyers” or “time wasters” alongside recognized moral tensions in making such distinctions. However, interviewees were quick to distinguish their own morally sanctioned use of services from the irresponsible behaviour of others:

P12: I was scared about my breathing and the pain because I'd never experienced anything like that. And, I wouldn't do it, you know, lightly. I mean, when you hear these horrendous stories about people going in. I heard it on the radio last week, on Radio Four. People going in to A&E for dandruff, for God's sake, you know. What is the matter with people? (Older interviewee)

Moral discourses were used when discussing the behaviour of others in a manner which sought to understand reasons for service use to defend their own behaviour. While the backdrop to this might be wider debates about service pressures and appropriate use, it seems that sense‐making is rendered problematic because of the lack of clarity regarding the boundaries between urgent and emergency health‐care services, and highly contingent nature of health‐care need.

### 3.4 Re‐imagined borders of urgent and emergency provision

Panel members were asked to consider the “Keogh model”^1^ and to suggest services that should be included, and discuss the confusion about access routes. All noted problems with having A&E/ED at the base of the diagram in a bold colour (red) because it drew attention to this service and seemed to emphasize its importance. Asked to redraw the diagram to match their own understandings the panels’ pictures looked different. Rather than using relational language to describe their revised models (eg, “less urgent than 999”) they wanted clearer information about what different services did to inform their sense‐making, and examples of the kinds of illness and injury that would be treated at each. Some drew traffic lights or representations that helped make sense of where to go:

The East European community panel provided particular insights into use of different services. Members noted not trusting phone services (such as NHS111) and preferred to see a doctor face to face, prompting them to seek help at the A&E.

We don't trust phone calls. We don't use them. Quite often, we don't communicate well enough to explain what's happening and take the message from the doctors on the phone. They don't have Polish speakers or any other languages, you know, on the 111. So that's why they don't call and they would like to see a doctor, because the doctor will explain. If they cannot explain, they draw it or they show it on a picture. So then she knows (East European panel)

Sense‐making of service use was imbued with the process, common in managing health and illness more generally, of establishing oneself as an appropriate candidate for using urgent care by emphasizing control over personal decisions, autonomy and independence, and being stoical in the face of adversity.^46^ Thorogood^47^ identified that Black African Caribbean women in Britain used private medicine to regain equality and power in this area of their lives (p. 35) and a similar claim may be made about the East European participants’ arguments marshalled in the panels, which legitimize decisions to use a particular service as a means of ensuring equality. Other participants in this panel mentioned direct experiences of racism as influencing choices about accessing care which support this interpretation. Experiences of health‐care systems elsewhere, and limited knowledge of the NHS, added to their confusion about the “map” of service provision alerting us to the need to consider cultural differences in sense‐making.

## 4 CONCLUSIONS

The demand for urgent and emergency care services appears to be increasing, especially from particular groups of patients who share characteristics of those purposively chosen in this study.^48^, ^49^ Our exploration of peoples’ sense‐making, experiences and views of the distinctions between urgent and emergency care suggests that boundaries between services are ill‐defined creating confusion about the appropriate use of the many services on offer. This may explain peoples’ difficulties navigating the use of services in ways officially considered “appropriate” as it makes sense from a service user's perspective to see boundaries with a fluidity not intended by policy. While participants acknowledged that health‐care needs were highly contingent, their sense‐making included a moral component which tended to be judgemental and polarized between one's self (help seeking is legitimate) and others (help seeking is inappropriate, unless there are special factors to take into account).

Previous research literature and wider policy rhetoric has revolved around or at least made use of this moral positioning, sparking media debates and atrocity stories about inappropriate attendance. Yet, this continued focus on help‐seeking behaviour, rather than that of sense‐making, makes it difficult to move beyond blaming the service user after they have attended. As a result policy, professionals and the public often simply polarize behaviours as “appropriate” and “inappropriate” service use. A deeper analysis of sense‐making, as we have shown, may shift the focus of attention and allow us to intervene and reshape understandings before this point. The implications of our analysis are twofold. First, that there needs to be a re‐imagining and clearer articulation of the model of service provision and of the differences between urgent and emergency care. The almost continual reconfiguration and extension of urgent and emergency care services has created considerable confusion which hinders sense‐making and may encourage service use that is labelled as inappropriate. Work with service users and citizens will be vital in developing language, definitions and models that will address the gaps in understanding and support better sense‐making to ensure that care and service use is optimized. Second, our study, by deliberately looking at groups of people drawn from populations with known high use of emergency care, and who might be considered more marginal, has highlighted a strong moral thread running through their accounts of help seeking and service use. It is not that people deliberately make “wrong” choices of about service use, but rather that their choices are socially constructed, and contingent, and informed by beliefs and experience. Different groups of users and citizens may draw on different understandings and knowledge and this may require more tailored and differentiated support for sense‐making.

## CONFLICT OF INTEREST

Catherine Pope, Gemma McKenna and Anne Rogers are members of the NIHR CLARHC Wessex.

## ETHICAL APPROVALS

The citizen panel element of the project received ethical approval from the University of Southampton Ethical Committee (Reference 20217). HRA ethical approval was obtained (REC reference number 16/EM/0329) for the semi‐structured interviews.
